# Monitoring of Persons with Risk for Exposure to Ebola Virus Disease — United States, November 3, 2014–March 8, 2015

**Published:** 2015-07-03

**Authors:** Tasha Stehling-Ariza, Emily Fisher, Sara Vagi, Ethan Fechter-Leggett, Natasha Prudent, Mary Dott, Randolph Daley, Rachel Nonkin Avchen

**Affiliations:** 1 Epidemic Intelligence Service, CDC; 2 Division of State and Local Readiness, Office of Public Health Preparedness and Response, CDC; 3 Oregon Public Health Division; 4 Division of Environmental Health Hazards and Health Effects, National Center for Environmental Health, CDC

On October 27, 2014, CDC released guidance for monitoring and movement of persons with potential Ebola virus disease (Ebola) exposure in the United States (1). For persons with possible exposure to Ebola, this guidance recommended risk categorization, daily monitoring during the 21-day incubation period, and, for persons in selected risk categories, movement restrictions. The purpose of the guidance was to delineate methods for early identification of symptoms among persons at potential risk for Ebola so that they could be isolated, tested, and if necessary, treated to improve their chance of survival and reduce transmission. Within 7 days, all 50 states and two local jurisdictions (New York City [NYC] and the District of Columbia [DC]) had implemented the guidelines. During November 3, 2014–March 8, 2015, a total of 10,344 persons were monitored for up to 21 days with >99% complete monitoring. This public health response demonstrated the ability of state, territorial, and local health agencies to rapidly implement systems to effectively monitor thousands of persons over a sustained period.

Enhanced entry screening was conducted at five U.S. international airports at which travelers from Ebola-affected West African countries were identified and assigned a risk categorization for Ebola exposure. The Ebola-affected West African countries and the U.S. risk categories have changed over time, as described in the CDC interim U.S. guidance (1). Enhanced entry screening identified symptomatic travelers needing further evaluation. Federal authorities screened, educated, and collected information on travelers. Traveler information was provided to state, territorial, and local public health authorities to conduct health monitoring (2). Health care workers (HCWs) who cared for Ebola patients domestically, including laboratory staff, were identified through their health care facilities. Guidance for monitoring and movement of persons with potential Ebola exposure recommended risk stratification and public health actions for each category (1). Four risk categories were created: high, some, low but not zero (in this report referred to as low), and no identifiable risk.*

After potential exposure to Ebola, one of two daily public health actions, either active monitoring (AM) or direct active monitoring (DAM), was required for 21 days. AM was recommended for low-risk travelers and consisted of twice-daily temperature checks and self-evaluation for symptoms consistent with Ebola (1,3). Persons under AM reported their health status to the public health authority overseeing monitoring at least once daily (1,4). DAM was recommended for persons at high risk or some risk, as well as for HCWs at low risk who had cared for Ebola patients in the United States. In addition to AM requirements, DAM included twice-daily reports to the monitoring jurisdiction with at least once-daily direct visualization of the individual by the health authority (1,4).

Complete monitoring (either AM or DAM) was defined as making contact with the monitored person with no gaps in reporting (e.g., no loss to follow-up) of >48 hours. Weekly estimates of the number of persons under monitoring and reporting symptoms, and calculations of incomplete monitoring were collected from the jurisdictions’ weekly reports. The overall estimate of persons under monitoring was calculated as the sum of persons reported as 1) completing monitoring, 2) leaving the United States during their monitoring period, and 3) remaining under monitoring on March 8, 2015.

Monitoring was conducted by 60 jurisdictions: the 50 states, NYC and DC, five U.S. territories (American Samoa, Commonwealth of the Northern Mariana Islands, Guam, Puerto Rico, and U.S. Virgin Islands), and three freely-associated states (Federated States of Micronesia, Republic of the Marshall Islands, and Republic of Palau) (4). Until March 9, jurisdictions submitted individual-level, daily reports to CDC for all persons under monitoring who were at high risk or some risk. These reports included data on monitoring (e.g., compliance and reported symptoms), transportation plans should the person become symptomatic, assigned assessment hospitals, and intrastate and interstate travel plans of persons under monitoring. All jurisdictions submitted aggregate weekly reports for persons at low risk (including reports when no one was monitored) and reported the same monitoring data as in the daily reports. Information on returning Department of Defense personnel restricted to a military station for their 21-day monitoring period was not reported to CDC and is reported elsewhere (5).

During November 3, 2014–March 8, 2015, in the 60 jurisdictions, 10,344 persons were monitored (Table). Overall, 91.9% of the persons monitored were travelers at low risk, 5.1% were HCWs at low risk who had provided patient care in the United States, and 3.0% were persons at high or some risk (Figure 1).

During the study period, a median of 1,710 persons (range = 1,331–2,119) were monitored in a given reporting week (Figure 2). Among HCWs at low risk caring for patients in the United States, 96% were monitored during November and early December, after giving care to the first patients treated for Ebola in the United States. In mid-December and early February, the number of persons at high risk or some risk increased 240% and 307%, respectively, corresponding with the return of two teams of U.S. Public Health Service officers who had staffed an Ebola treatment unit in Monrovia, Liberia.

In a given week, a median of 1.5 persons for whom monitoring was indicated could not be contacted upon arrival in the jurisdiction (0.4%; range = 0–48 persons per week). The number of persons who could not be contacted in a given week decreased from a median of 23 persons per week (1.4%) in November to less than one person per week in February (0.03%). Of the persons ever contacted for monitoring, a median of 7.5 persons had gaps in being monitored that were >48 hours in a given week (0.6%; range = 1–26 persons per week). The median number of persons with >48-hour gaps in monitoring decreased from 20 persons per week (1.0%) in November to three per week (0.2%) in February.

During a given reporting week, a median of 20 persons under monitoring (1.2%, range = 9–43 persons) reported Ebola-compatible symptoms. The number of symptomatic persons peaked in December 2014. Of the symptomatic persons in the low-risk and some-risk categories, 39 were tested for Ebola during their monitoring period; none tested positive for Ebola. No persons at high risk reported Ebola-compatible symptoms.

All 50 states, DC, NYC, Puerto Rico, and the U.S. Virgin Islands monitored persons at low risk (Figure 3). Forty-four states, DC, NYC, and Puerto Rico monitored one or more persons at high risk or some risk. Three territories and three freely-associated states had no persons under monitoring. More than half (54%) of the persons were monitored in five jurisdictions. The most persons were monitored in NYC, followed by Maryland, Pennsylvania, Georgia, and Virginia (Figure 3). NYC monitored nearly twice as many persons as Maryland.

## Discussion

Within 7 days of issuance of CDC guidance on movement and monitoring in October 2014, all 50 states and two local jurisdictions were effectively monitoring travelers arriving from countries with widespread Ebola transmission and HCWs caring for patients with Ebola in the United States. By December 22, all U.S. territories were reporting to CDC. Less than 1% of monitoring was incomplete. Anecdotally reported reasons for incomplete monitoring included missing or incorrect contact information, logistical issues (e.g., transfer from one jurisdiction to another), and noncompliance by persons being monitored.

These efforts demonstrate the capacity and infrastructure developed by U.S. jurisdictions to urgently respond to a large-scale monitoring need. Since 2002, considerable resources have been distributed to public health departments to effectively respond to infectious disease outbreaks and other public health threats (6). Additional resources also have been awarded to jurisdictions for Ebola-related activities.

The findings in this report are subject to at least two limitations. First, because weekly data were reported in aggregate, the estimated numbers of persons monitored might be inexact. For example, overestimates would result if a jurisdiction reported the same person in both low-risk and some-risk categories for a given reporting period. This likely would occur when a person’s risk classification changed during the 21-day monitoring period (e.g., an HCW who completed work in an Ebola treatment unit days before departing the country could change from some risk to low risk). Duplicates were corrected whenever identified. Second, the calculation of the overall number of persons under monitoring might be an underestimate if all persons were not reported as having completed their monitoring, leaving the United States, or still being under monitoring on March 8, 2015.

These results provide evidence of successful U.S. monitoring for Ebola. Jurisdictions demonstrated public health capacity to rapidly conduct and effectively monitor thousands of persons over a sustained period. After monitoring of 10,344 persons, no transmission of Ebola was reported during the study period, and few persons under monitoring reported symptoms suggesting potential Ebola infection (7). Given the complexity and amount of coordination of effort required, the Ebola monitoring program in the United States provided systemic evidence of the capability of state, territorial, and local health departments to ensure and protect the health of the U.S. public.

**Summary**

What is already known on this topic?

The 2014–2015 Ebola virus disease (Ebola) epidemic is the largest ever reported. During March 25, 2014–June 23, 2015, a total of 15,109 laboratory-confirmed cases of Ebola were reported and 11,232 persons died, primarily in Guinea, Liberia, and Sierra Leone. To prevent transmission of Ebola in the United States, CDC issued monitoring and movement guidance on October 27, 2014, and provided epidemiologic and clinical expertise in support of 60 jurisdictions’ implementation of this guidance.

What is added by this report?

This report is the first to present results from the 60 U.S. jurisdictions that monitored persons with potential exposure to Ebola, including those returning from Ebola-affected countries. A total of 10,344 persons were monitored during November 3, 2014–March 8, 2015, with >99% complete monitoring.

What are the implications for public health practice?

This report provides evidence that jurisdictions can rapidly implement a complex monitoring system and monitor thousands of persons with potential exposure to Ebola over a sustained period. In addition, this report provides documentation that among the 10,344 monitored, none were diagnosed with Ebola.

## Figures and Tables

**FIGURE 1:**
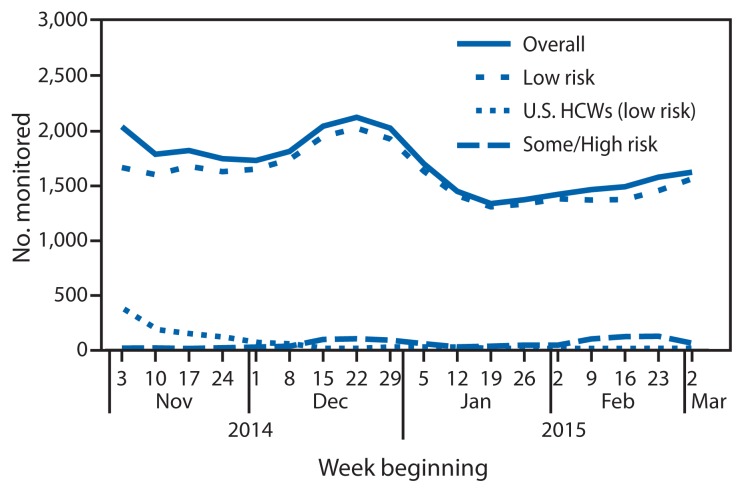
Number of persons (N = 10,344) with potential Ebola exposure who were monitored, by risk category and week — United States, November 3, 2014–March 8, 2015 **Abbreviation:** HCWs = health care workers.

**FIGURE 2:**
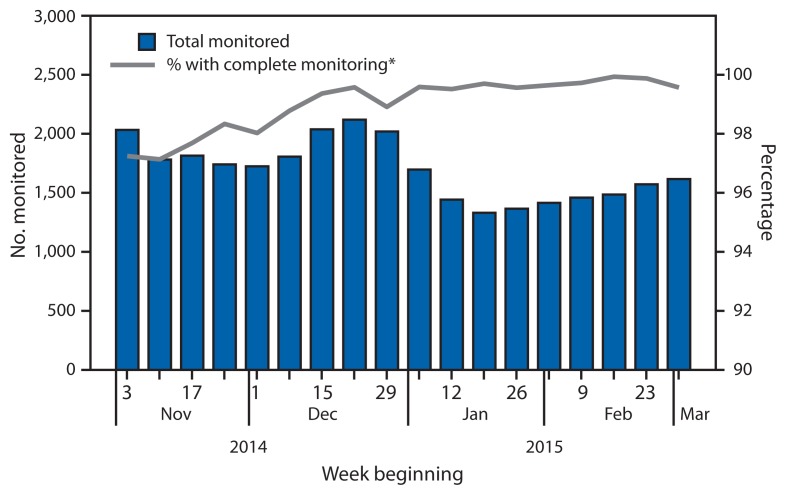
Number of persons (N = 10,344) with potential Ebola exposure who were monitored and percentage with complete monitoring, by week — United States, November 3, 2014–March 8, 2015 * Complete monitoring is defined as making contact with the monitored person with no gaps in reporting of >48 hours.

**FIGURE 3:**
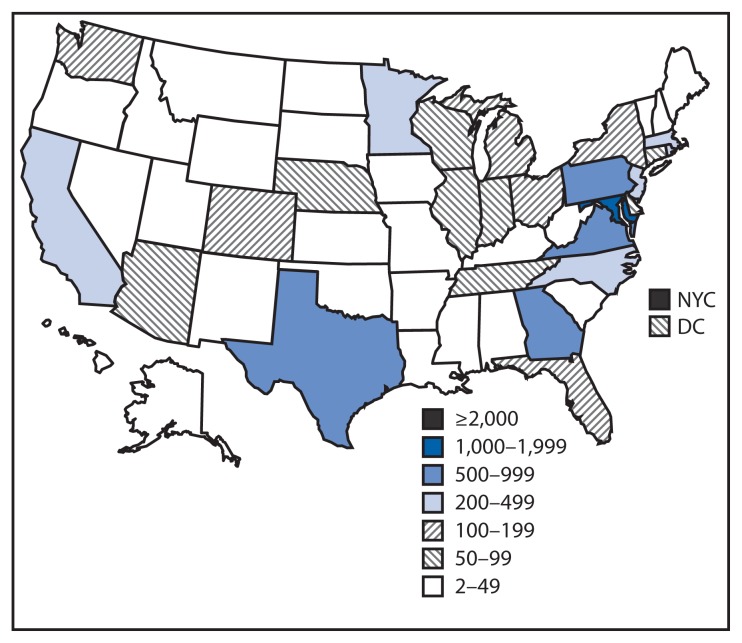
Number of persons with potential Ebola exposure monitored in 50 states, New York City, and the District of Columbia — November 3, 2014–March 8, 2015

**TABLE:** Summary of active and direct active monitoring of persons with potential Ebola exposure, by risk category — United States, November 3, 2014–March 8, 2015

	Risk category			
		
		Low (but not zero) risk		
			
Monitoring element	High risk and some risk	Travelers	U.S. HCWs	Total
Type of daily monitoring	DAM	AM	DAM	—
Reporting frequency to CDC	Daily	Weekly	Weekly	—
No. of persons monitored	315	9,512	527	10,344*
No. of jurisdictions conducting monitoring	47	54	10	54

**Abbreviations:** AM = active monitoring; DAM = direct active monitoring; HCWs: Health care workers, including laboratory personnel.

* Adjusted for persons whose risk category changed from some risk to low risk.

## Footnotes

* Additional information available at http://www.cdc.gov/vhf/ebola/exposure/risk-factors-when-evaluating-person-for-exposure.html.
